# Specific Expression of DR5 Promoter in Rice Roots Using a tCUP Derived Promoter-Reporter System

**DOI:** 10.1371/journal.pone.0087008

**Published:** 2014-01-22

**Authors:** Jie Zhou, Feibo Yu, Xuming Wang, Yong Yang, Chulang Yu, Hongjia Liu, Ye Cheng, Chengqi Yan, Jianping Chen

**Affiliations:** 1 State Key Laboratory Breeding Base for Zhejiang Sustainable Pest and Disease Control, MOA Key Laboratory for Plant Protection and Biotechnology, Zhejiang Provincial Key Laboratory of Plant Virology, Zhejiang Academy of Agricultural Sciences, Hangzhou, P. R. China; 2 College of Chemistry and Life Sciences, Zhejiang Normal University, Jinhua, P. R. China; Iwate University, Japan

## Abstract

Variation of transgene expression caused by either position effect at the insertion site or the promoter/enhancer elements employed for the expression of selectable marker genes has complicated phenotype characterization and caused misinterpretation. We have developed a reporter system in rice to analyze the influence of vector configuration, spacer and selectable marker gene promoter on the expression of the promoterless GUS reporter and DR5 promoter. Our results indicate that a spacer inserted between the reversed 35S promoter and the GUS reporter could reduce leaky expression of the reporter but was unable to block the nonspecific expression of DR5::GUS. Stacking the selectable marker unit in head to tail with the GUS reporter aided the gene specific expression of the GUS reporter under the DR5 promoter even when the 35S promoter is used for expression of the selectable marker. Compared to 35S under this configuration, a quick and distinctive expression of DR5::GUS was observed in the root cap, quiescent center and xylem cells in the root apical meristem by using the tCUP derived promoter (tCUP1) for selection, that is similar to the pattern obtained by a sensitive DR5 variant (DR5rev) in *Arabidopsis*. These data suggest a conserved property of the tCUP promoter in preventing enhancer-promoter interactions in rice as it does in *Arabidopsis*, and also demonstrate that an analogous distal auxin maximum exists in roots of rice. Therefore, the tCUP promoter based selection system provides a new strategy for specific expression of transgenes in rice.

## Introduction

To study gene function and genetic engineering in plants it is important to have precise expression of transgenes, but differences in transgene expression patterns have often been observed between independent transformants [1]. They are generally ascribed to local modulations from the flanking DNA at the different integration sites leading to position effects [2], but expression of transgenes may also be influenced by the transgene construct introduced. As an efficient selection procedure is a prerequisite for successful plant transformation, a number of widely used transformation vectors for both dicots and monocots use the single or double 35S promoter from cauliflower mosaic virus (CaMV) to achieve high throughput and effective selection of transformants [3]–[6]. These vectors include the pCAMBIA series (http://www.cambia.org/daisy/cambia/materials/vectors.html), pPZP family vectors, pINDEX1 and Gateway’s pGWB. However, experiments with these vectors have shown that the strong 35S promoter used to drive the expression of the selectable marker gene causes significant interference in the strength and specificity of adjacent promoters, especially those with distinctive spatiotemporal activities [7], [8]. This distorted pattern of expression may be exacerbated in analyses of truncated or synthetic promoters that lack the natural insulating sequences of the native promoter [9]. An increasing awareness of this drawback has stimulated investigations into the mechanisms underlying the enhancer-mediated multi-promoter interactions, as well as strategies to avoid or prevent them [10].

The first type of strategy is to separate the promoter of the selectable marker gene from that of the target gene. To achieve this purpose, the most desirable approach is to separate the transcriptional gene units in the plant genome by the co-transformation method. In such a way, the selectable marker gene unit, or only the promoter for the selectable marker gene, is not included in the same T-DNA as the target gene controlled by its own specific promoter. This non-selectable vector can then be co-transformed with the selectable vector by mixing two *Agrobacterium* strains each containing one of the vectors or by a single strain containing both vectors. However, the percentage of transformed plants carrying both T-DNAs integrated at different loci was relatively low in rice [11], necessitating the creation and screening of a large number of transgenic lines for a particular transgene.

Another approach to separating the two promoters relies on the configuration of the expression units within the same T-DNA. The promoters can be separated by stacking the expression units in head to tail or tail to tail orientation, while in most vectors they are stacked head to head. However, no detailed comparison of these configurations has been made in terms of their effects on transgene expression, except that we know that the strong position effects from the integration site would override the expression of the transgene located proximal to the T-DNA border, which has been used for promoter or enhancer trapping experiments [12], [13]. As an alternative, the distance between the expression units could be increased by incorporating a spacer fragment thus reducing interference, but the precise distance required to block the enhancer mediated interaction varies with different enhancer/promoter combinations [14], [15].

The second type of strategy uses alternative promoters that do not affect the specificity of nearby promoters. Replacement of 35S with promoters derived from *A. tumefaciens*, such as nopaline synthase (*nos*) [16] or mannopine synthase (*mas*) [17] can reduce the ectopic expression of transgenes in some cases [8], [15]. Among the plant derived promoters, the tobacco cryptic promoter (tCUP) [18] and its enhanced modifications are highly active in a wide range of plant species, and exhibit activities exceeding those of the 35S promoter [19], [20]. Subsequently tCUP has been successfully used for selectable marker gene expression [14], [21], and did not interfere with some seed-specific promoters in *Arabidopsis* regardless of T-DNA configuration and distance between the two promoters [14]. Recently we reported that the tCUP derived promoter tCUP1 can also drive selectable marker gene expression in rice, although its activity is much lower than that of the 35S promoter [22]. Thus it would be interesting to know whether the tCUP1 promoter has the similar property for stringent regulation of gene expression in rice.

Finally the last promising strategy proposed to mitigate enhancer-promoter interactions is flanking transgenes with insulators or boundary DNA elements as they can block the effects of neighboring enhancers and silencers as well as encroaching heterochromatin [23]. Although a number of different DNA sequences with insulating or boundary activity have been identified in vertebrates [24]–[28], most of them proved abnormal or ineffective in plants [14]; only the *gypsy* insulator of *Drosophila* was reported to facilitate high and precise transgene expression in *Arabidopsis* [29]. So far, only two sequences from plants have been identified as enhancer-blocking insulators [30], [31]. Unfortunately, one of them, the *Arabidopsis NI29* fragment, failed to protect the adjacent promoters from activation by the 35S enhancer in further studies [14], [32]. Nevertheless, the transformation booster sequence (*TBS*) MAR from petunia was found operational in both *Arabidopsis* and tobacco [33].

Auxin is a key hormone in plant morphogenesis influencing cell division, elongation and differentiation. Polar auxin transport and gradient auxin distribution are necessary for correct distal pattern formation in plants, including early embryogenesis and root development [34]–[38]. The most widely used tool to visualize auxin distribution is the synthetic DR5 based auxin inducible reporter whose promoter contains seven tandem repeats of an auxin-responsive TGTCTC element [39]. Using the DR5 reporter, auxin distribution and signaling responses have been well-documented in *Arabidopsis*, for example the distal auxin maximum localization in the root meristem [35], [40]. The same procedure has since been applied to many other plant species, including rice [36], [41]–[45]. However, there has not been a comprehensive study of the expression patterns of DR5 in rice, and strong expression in the whole stele often occurred in currently used DR5::GUS lines [46], [47]. Although they have been widely used in auxin signaling studies because their responsiveness to auxin was not affected [46], [48], studies of auxin transport and localization in rice may be problematic with these lines. We consider that may largely result from side effects of the transgene construct, especially the nearby 35S promoter within the vector mostly used. To test this hypothesis, DR5::GUS reporter vectors were constructed in combination with three different selectable marker promoters and four stacking configurations. By comparison of expression patterns in the root tips of transgenic plants, we found that the DR5 promoter was distinctively expressed in similar regions of rice root tips as it is in *Arabidopsis*, but that such a specific pattern was mainly obtained by a vector in head to tail tandem configuration with a tCUP derived promoter (tCUP1) close to the T-DNA left border. We also demonstrated that examples of strong DR5::GUS expression in the entire stele was nonspecific expression activated by the adjacent 35S promoter, whereas with an adjacent tCUP1 promoter there was weak expression in the xylem. Thus the tCUP derived promoter was able to reduce nonspecific interactions in rice. Combined with configuration analysis, we suggest a promising vector system to achieve specific transgene expression in rice.

## Results

### A Promoterless Reporter System to Study the Mis-expression Effect Caused by Promoter Activity, Spacer and Configuration

**Figure 1:**
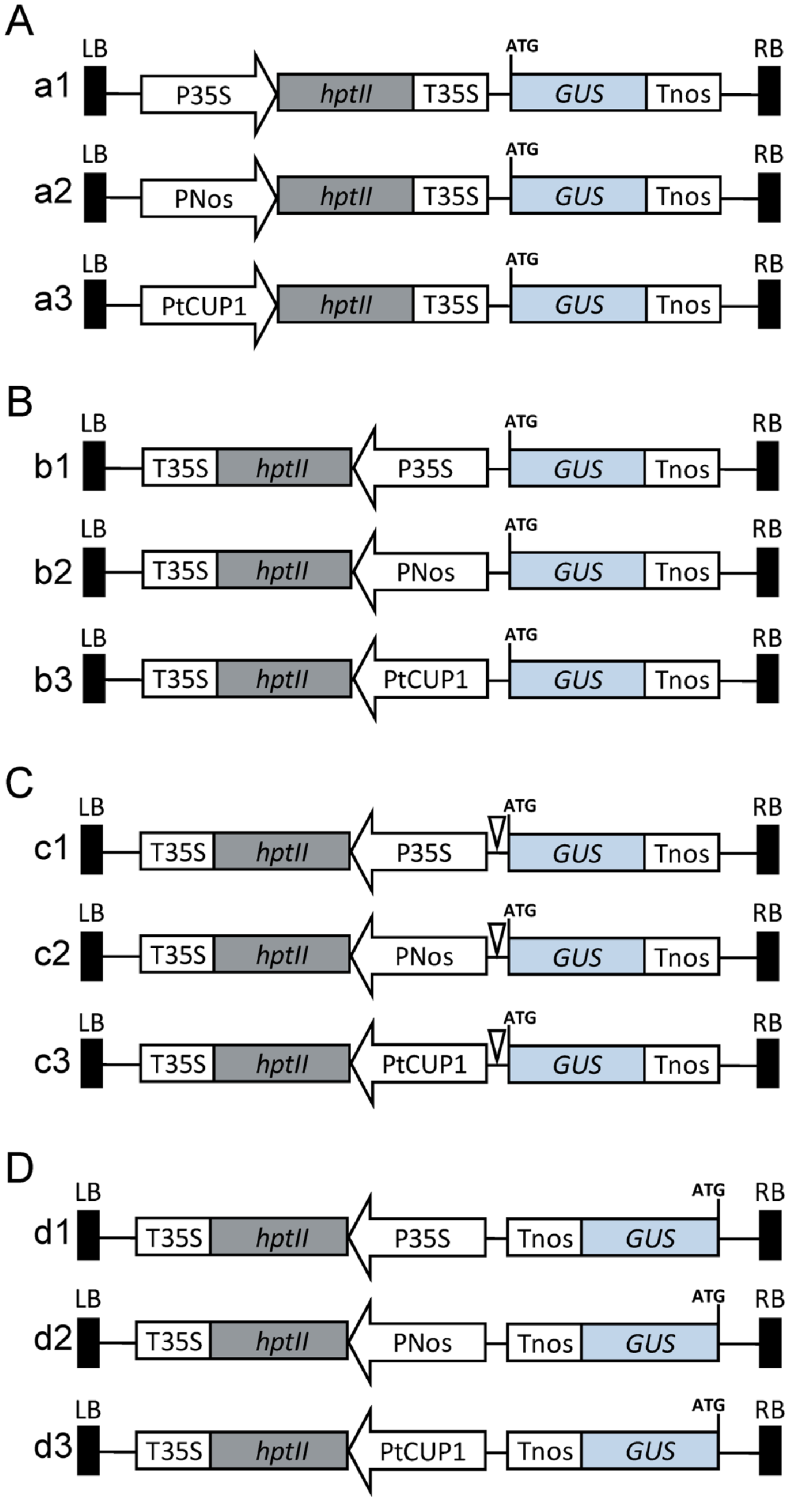
T-DNA constructs created to combine promoterless GUS reporter vectors with different configurations and promoters to express selectable markers. (A) Group of vectors constructed in head to tail orientation with selectable marker upstream of the GUS reporter. The selectable marker cassette is comprised of hygromycin phosphotransferase gene (*hptII*, 1026 bp, in gray box) driven by CaMV 35S (848 bp, a1), Nos (307 bp, a2) or tCUP1 (519 bp, a3) promoters and terminated by the same CaMV 35S terminator (225 bp). The β-glucuronidase reporter gene (GUS, 2053 bp, in blue box) with nopaline synthase (nos) terminator (268 bp) at the 3′ end near the T-DNA right border (RB). (B) Group of vectors constructed in head to head orientation with reversed selectable marker upstream of the GUS reporter. The selectable marker gene is differentially expressed by CaMV 35S (b1), Nos (b2) or tCUP1 (b3) promoters placed near the start codon of GUS reporter. (C) Group of vectors constructed in head to head orientation similar to B series vectors except that a 254 bp sequence (triangle) derived from pCAMBIA1300 was inserted to separate the GUS reporter and its adjacent inverted promoter used for *hptII* gene expression. (D) Group of vectors constructed in head to tail orientation with GUS reporter upstream of the selectable marker. The GUS reporter gene is stacked with ATG near the RB and nos terminator close to CaMV 35S (d1), Nos (d2) or tCUP1 (d3) promoters used for *hptII* gene expression.

To precisely monitor the transcript level outputted by any *cis* regulatory element in rice via a reliable reporter vector, a promoterless reporter system was developed to analyze the likelihood of mis-expression caused by the flanking sequences. Four sets of vectors were designed in three orientations that cover the relative relationships between cassettes of selectable marker and reporter genes in tandem (Fig. 1A and 1D) or in head to head (Fig. 1B and 1C) configuration, and reporter outward (Fig. 1D) or inward (Fig. 1A, 1B and 1C) relative to the T-DNA border. To test the effect of distance between two cassettes in head to head orientation, a 254 bp fragment from pCAMBIA1300 (between the CaMV 35S promoter and multiple cloning sites) was inserted before the promoterless GUS reporter as a spacer (Fig. 1C). In each set of vectors, three promoters with different activities [22] were used to drive selectable marker gene expression and to investigate the interaction probabilities between these tested promoters and the GUS reporter. The promoters used were the 35S promoter of cauliflower mosaic virus (CaMV 35S, Fig. 1, a1–d1), *Agrobacterium* nopaline synthase promoter (Nos, Fig. 1, a2–d2) and tobacco cryptic promoter (tCUP1, Fig. 1, a3–d3).

**Figure 2:**
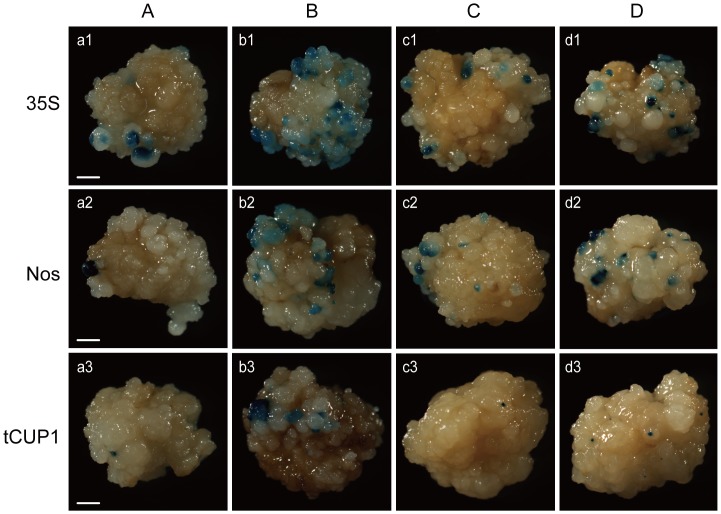
GUS leaky expression in calli transformed by promoterless GUS reporter vectors containing different selectable marker promoters in different stacking configurations. GUS leaky expression in representative callus transformed by promoterless GUS reporter vectors in (A–D) configurations combined with 35S (a1,b1,c1,d1), Nos (a2,b2,c2,d2) or tCUP1 (a3,b3,c3,d3) selectable marker promoters as indicated in Figure 1. Fifty Transformed calli per vector were stained after 3 weeks selection and calli with the average numbers of staining spots were pictured individually at the same magnification. Pictures show representative calli for at least three replicates. Bars: 1 mm.

**Figure 3:**
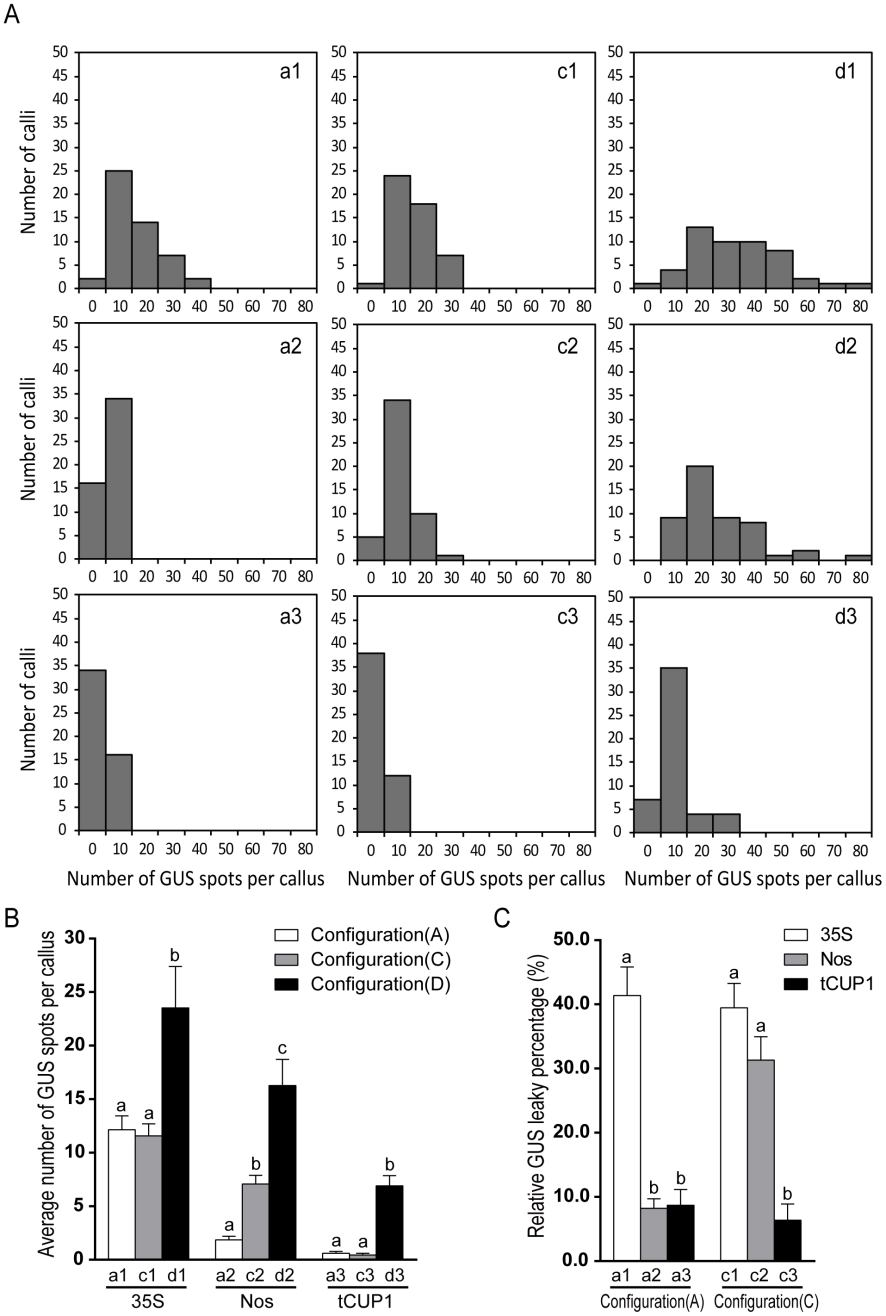
Comparison of GUS leaky expression in calli among vectors with different selectable marker promoters in different stacking configurations. (A) Distribution histograms showing the numbers of spots of GUS leaky expression in calli transformed by group A (a1–a3), C (c1–c3) and D (d1–d3) vectors. Numbers of GUS spots per callus was set in the range from 0 to 80 and divided into 9 intervals. A total of 50 calli were counted for each vector. (B) Average number of GUS spots per callus derived from each vector. The bar represents mean±SE of spot numbers from 50 calli counted in (A). Different letters above the column indicate a statistically significant difference (p<0.05) between vectors with the same selectable marker promoter. (C) Relative GUS leaky percentage between group A (a1–a3) and group C (c1–c3) vectors after normalization by average spot numbers per callus of the respective group D (d1–d3) vectors. Different letters above the column indicate a statistically significant difference (p<0.05) between vectors with the same configuration. Statistical analysis was performed with GraphPad Prism 6 software (GraphPad Software Inc., La Jolla, CA) by using one-way ANOVA with a Tukey’s multiple comparisons test.

All four groups of vectors (Fig. 1) were transformed to rice calli using *Agrobacterium*, and GUS leaky expression was determined by histochemical staining of calli after 3 weeks of selection. Various extents of leaky expression were observed between different combinations of stacking configuration and promoter used for selectable marker expression (Fig. 2). Among these combinations, the group B vectors have the maximum number of GUS expressing spots on the surface of calli (Fig. 2, b1–b3), which may be attributed to the inverted selectable marker promoter being close to the GUS reporter. Compared to Nos and tCUP1 promoters, the inverted 35S CaMV activated the strongest GUS expression as expected from its enhancers (Fig. 2, b1). Progressively reduced, but still obvious, GUS expression was observed in B group vectors containing Nos and tCUP1 (Fig. 2, b2 and b3), which suggested that bidirectional activity also occurs in these two promoters. To decrease the influence of bidirectional promoter activity on GUS expression, a 254 bp fragment from the pCAMBIA1300 vector which separates the 35S promoter and multiple cloning sites was inserted between the inverted promoter and GUS reporter. A great reduction in GUS expression occurred with all three promoters (Fig. 2, c1–c3). Notably, the GUS expressing spots were dramatically reduced to a single digit level after the tCUP1 promoter was separated by the spacer (Fig. 3A, c3). These results indicated that the reverse bidirectional promoter activity could be effectively inhibited by increasing the distance to its nearby expression cassette, especially for the tCUP1 promoter. We therefore wanted to know whether the distance would also decrease the activity of the forward promoter in its interaction with the GUS reporter. When the selectable marker cassette was stacked upstream of the GUS reporter in head to tail orientation (Fig. 1A, a1–a3), the promoter and the GUS reporter was then separated by the selectable marker and its 3′ terminator. Similar leaky expression was observed for the 35S promoter in both spaced forward and reverse stacking orientations (Fig. 2, a1 and c1; Fig. 3, a1 and c1). The same situation also occurred with the tCUP1 promoter; only single numbers of GUS spots could be seen in both spaced configurations (Fig. 2, a3 and c3; Fig. 3, a3 and c3). In contrast, the Nos promoter in forward configuration had much lower interactions with the GUS reporter than in reverse configuration with spacer (Fig. 2, a2 and c2; Fig. 3, a2 and c2). These data suggested that long range activation of the GUS reporter is also limited when the selectable marker is configured upstream of the reporter in head to tail orientation. The last configuration tested placed the promoterless GUS gene close to the T-DNA border (Fig. 1D, d1–d3). As when a similar structure was used for promoter trapping experiments, many, but countable, GUS staining spots were observed, especially in vectors with the selectable marker driven by 35S and Nos promoters (Fig. 2, d1–d3; Fig. 3A, d1–d3). This abundant GUS leaky expression suggested that the influence of the position effect at the insertion site was stronger than the interaction emanating from the selectable marker promoter in the other two spaced orientations (Fig, 3B). As previously reported the promoter activity was different between 35S, Nos and tCUP1 which will influence the number of transformed cells [22]. The GUS spot numbers obtained by D group vectors may partially correlate with the total number of cells transformed because of random insertion of T-DNAs. For further comparisons between vectors with different promoters within the same configuration, the average number of GUS spots per callus was normalized by the value obtained with the D group of vectors to minimize the influence of different transformation efficiency corresponding to each promoter. The resulting relative GUS leaky percentage in group A and C vectors suggested that the tCUP1 promoter was least likely to activate the transcription of the GUS reporter in both A and C configurations, whereas the 35S promoter is most prone to interact with GUS expression. Interestingly, low leaky percentage was found for the Nos promoter when in A type of configuration, comparable to that in vectors containing the tCUP1 promoter (Fig. 3C).

### Expression Patterns of DR5::GUS in Rice Roots with Different Reporter Vectors

**Figure 4:**
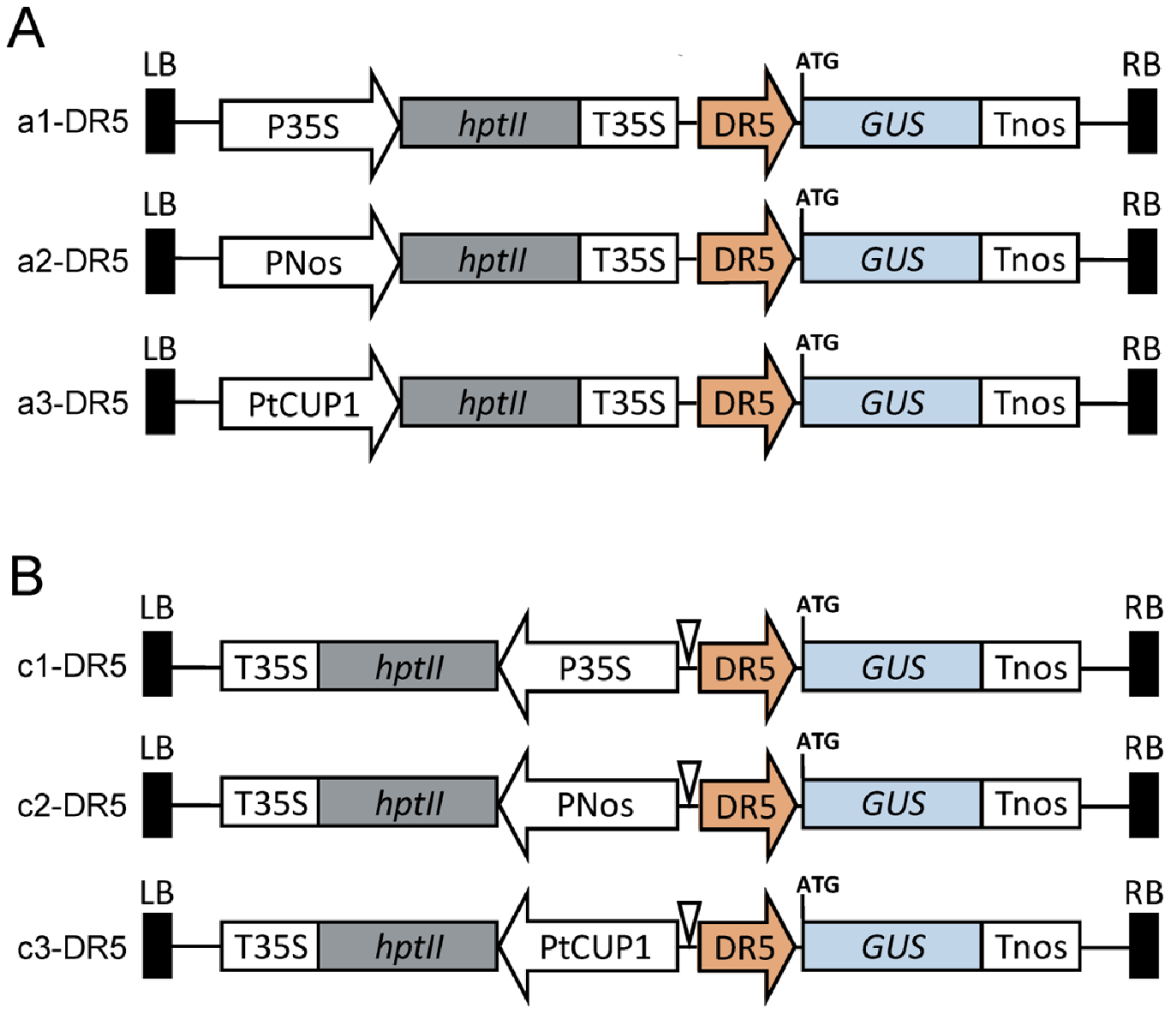
Construction of DR5::GUS reporter vectors on the basis of A and C configurations. (A) The synthetic auxin responsive promoter DR5 (267 bp, yellow arrow) was inserted in group A vectors before the GUS reporter to make a1-DR5, a2-DR5 and a3-DR5. (B) A similar strategy was used to construct the DR5 promoter in group C vectors and named c1-DR5, c2-DR5 and c3-DR5 respectively.

**Figure 5:**
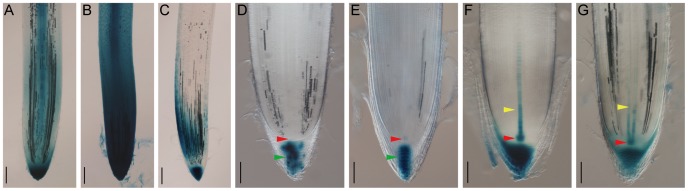
Specific and non-specific expression of DR5::GUS in adventitious roots of T_0_ transgenic plants. (A, B) Non-specific expression of DR5::GUS in root tip and stele in both weak (A) and strong (B) staining lines. (C) Staining pattern with DR5::GUS expressed in root cap and zone of elongation. (D, E) Specific localization of DR5::GUS in columella cells only (D) or in columella cells and quiescent center (E). (F, G) Specific expression of DR5::GUS in root cap, quiescent center and protoxylem cells in the root meristem. Roots positioned in a protophloem plane, xylem cells expressing GUS are arranged in a uni-cellular plate (F) or roots positioned in a protoxylem plane, two arrays of protoxylem cells expressing GUS (G) are shown. Bars: A–C, 200 µm, D–G, 100 µm. Adventitious roots of 1-month-old T_0_ plants were collected and stained in 1 mM X-gluc for 1 h (A–E) or 0.5 mM X-gluc for 4 h (F, G). Positions of columella cell (green arrow), quiescent center (red arrow) and xylem cell (yellow arrow) are indicated. Detailed organization of rice root tissue and cell types can be found in references [56], [57].

Based on the results of GUS leaky expression analysis, group A and C constructs were chosen for further study with the DR5 promoter as they were shown to have less mis-expression potential than B and D groups. Six DR5::GUS constructs were developed by cloning the DR5 promoter directly before the GUS reporter gene (Fig. 4), and subsequently transformed to rice. A total of 202 independent lines were recovered from transformations with the six vectors. The DR5::GUS expression patterns in roots were first analyzed by histochemical staining of the newly developed adventitious root tips of the transgenic T_0_ plants. From the 150 GUS staining positive lines, four major patterns of expression for DR5::GUS in root tips occurred regularly, including specific and non-specific expression (Fig. 5). The general non-specific expression pattern exhibited blue coloration in the entire root tip and whole stele extending to the elongation zone in both strong and weak expression lines (Fig. 5A and 5B). This type of pattern was most frequently observed in lines transformed with the c1-DR5 vector (41 out of 46), occasionally found in lines transformed with the c2-DR5 vector but did not occur in lines transformed with the other four vectors. The specific expression pattern showed clear blue staining in the root cap, central quiescent center and one-cell-thick layer of immature xylem cells (Fig. 5F and 5G). This typical pattern was found in lines transformed with the a3-DR5 vector which uses the tCUP1 promoter to drive selectable marker gene expression. Unlike the comprehensive GUS expression found in c1-DR5 transgenic plants, the specificity of DR5::GUS expression was increased when using the a1-DR5 vector where the forward 35S was separated by the selectable marker. This was demonstrated by the frequent distribution of DR5::GUS in the root columella derivative cells (Fig. 5D and 5E), partially similar to its expression in *Arabidopsis* where DR5::GUS is maximally expressed in the columella initials and their derivatives after a short time of staining [35]. Many of the other a1-DR5 lines also had expression at the junction of the meristematic region and the elongation zone except in the columella cells (Fig. 5C). These results strongly suggest that stacking the selectable marker and GUS reporter in A configuration was better for specific promoter analysis than the C configuration, even when using the 35S promoter.

**Figure 6:**
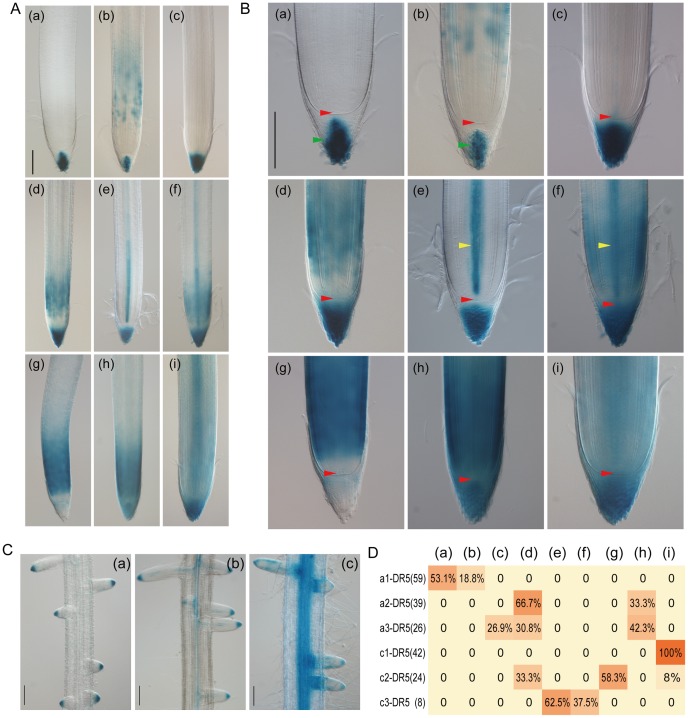
Summary of DR5::GUS staining patterns in primary roots of T_1_ transgenic plants. (A) Varied expression patterns of DR5::GUS (a-i) in primary root tips of 8-day-old T_1_ transgenic seedlings grown in IRRI rice solution. The most intense GUS expression was mainly observed in columella (a), in columella and zone of elongation (b), in root cap (c), in root cap and basal meristematic zone (d), in root cap and protoxylem (e), in root cap, protoxylem and meristematic zone (f), in basal meristematic and elongation zones, absent in root cap and promeristem (g), evenly stained in root cap and meristematic zone (h), in root cap, stele and meristematic zone (i). Samples were stained for 30 min for (c)-(i) or 16 h for (a) and (b). Bar: 100 µm. (B) Images (a-i) shown on the right are magnifications of the corresponding images shown on the left (A) to provide detailed staining patterns in the apical root. Positions of columella cell (green arrow), quiescent center (red arrow) and xylem cell (yellow arrow) are indicated. Bar: 100 µm. (C) Three major staining patterns in lateral roots (a-c) from all the analyzed T_1_ transgenic seedlings transformed by six DR5::GUS vectors. The most intense GUS expression was clearly observed in lateral root cap (a), in lateral root cap and xylem of both primary and lateral roots (b), in the whole vascular bundle and less specifically in the lateral root cap (c). Bars: 100 µm. (D) Patterns within T_1_ transgenic seedlings of each vector were classified and presented as a heat map of percentage. Number of GUS positive T_1_ seedlings observed for each vector is indicated in brackets.

A detailed analysis of the DR5::GUS expression patterns in primary roots was conducted in T_1_ transgenic plants transformed by the six DR5::GUS vectors. 272 T_1_ seedlings from 62 independent T_0_ lines were analyzed 8 days after germination by staining primary root segments from the root tip to the lateral root emerging region. Nine major staining patterns were observed in the primary root tips of the 198 GUS positive T_1_ plants (Fig. 6A and 6B). The frequency of these patterns with each vector is summarized in Fig. 6D. Two to three patterns were associated with each vector, except for c1-DR5 where all plants had the same pattern characterized by strong staining in the stele, similar to that of T_0_ (Fig. 5A, 5B and 6Ai). In the a1 configuration, DR5 directed specific GUS expression in columella cells of half the T_1_ plants (Fig. 6Aa), or in columella cells as well as the elongation zone (Fig. 6Ab). The remaining a1-DR5 plants had blue staining in lateral root tips but none in the primary root tip. In the a3 configuration, intense blue staining occurred in three types equally: root cap, root cap together with basal meristematic zone or evenly stained root cap and meristematic zone (Fig. 6Ac, 6Ad and 6Ah). A very weak expression in the quiescent center and xylem could be found in pattern c (Fig. 6Bc) but not clearly in patterns d and h (Fig. 6Bd and 6Bh), whereas in c3 configuration, xylem cells in the meristematic and elongation zones quickly responded to staining in addition to cells in the root cap (or root cap and meristematic zone) (Fig. 6Ae and 6Af). Notably, the quiescent center and vascular initials were not, or were only slightly, stained in these two patterns (Fig. 6Be and 6Bf). A few plants containing c2-DR5 had the non-specific staining pattern observed with c1-DR5 (Fig. 6Ai), but more than half of them had extensive blue staining only in the meristematic and elongation zones (Fig. 6Ag), a pattern never found with the other five vectors. By contrast, two patterns occurred when using the a2-DR5 vector, which were also found in plants with a3-DR5 (Fig. 6Ad and 6Ah). In spite of the variable expression patterns in the primary root tip, DR5::GUS expression was relatively stable in the lateral root region. Most of the plants derived from transformations of DR5 in A group vectors showed distinct GUS activity in lateral root caps (Fig. 6Ca), even those that had b, d or h patterns in their primary roots. Plants transformed by c1-DR5 also showed expression in parts other than the lateral root cap, especially a very strong expression in the stele of the main root (Fig. 6Cc). Plants transformed by c3-DR5 consistently had a pattern with clear expression in the lateral root cap as well as in the xylem of both lateral and primary roots (Fig. 6Cb). In addition, c2-DR5 plants which lacked staining in the primary root cap (Fig. 6Ag and 6Bg) also had no expression in the lateral root cap.

We can draw several conclusions from these observations of DR5::GUS expression in roots. Firstly, strong GUS staining of the whole stele was always found in plants transformed using a vector containing 35S in the C type of configuration, but was dramatically reduced when this promoter was replaced with Nos or tCUP1. This result suggests that the DR5 activity in the stele is augmented by the nearby 35S promoter in reverse orientation. Secondly, the specificity of DR5::GUS expression was increased when stacking the 35S promoter in configuration A, with GUS expression in columella cells, similar to the early staining pattern described for *Arabidopsis* [35]. This result together with that from Nos and tCUP1 indicated that A is the better configuration for specific promoter analysis. Thirdly, the typical DR5::GUS expression (in root cap cells, quiescent center and expanding to the xylem-precursor cells) was found when using the tCUP1 promoter, but not the Nos promoter, to drive selectable marker expression in A configuration, and was stronger than that with the 35S promoter. Thus, in this study, the best construct to analyze the DR5::GUS expression is a3.

### Auxin Responsiveness of the DR5 Promoter in Rice Roots

**Figure 7:**
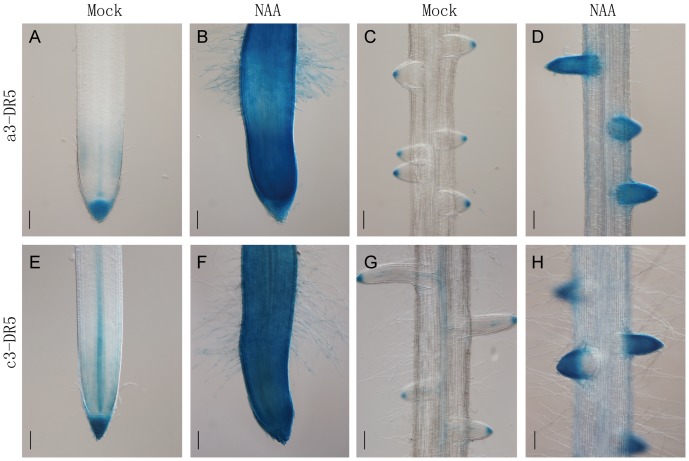
Auxin responsiveness in DR5::GUS transgenic lines selected by vectors with *hptII* controlled by tCUP1 promoter. Both a3-DR5 (A–D) and c3-DR5 (E–H) transgenic lines using the tCUP1 promoter for *hptII* gene expression were selected for the auxin responsive test. Representative images are shown for two independent lines each showing the DR5 promoter strongly induced in primary root tips (B, F) and lateral roots (D, H) after 1 µM NAA treatment for 24 h. In the mock treatment, specific but different DR5::GUS expression was observed between a3-DR5 and c3-DR5 lines in primary root tips (A, E) as well as in lateral roots (C, G). Bars: 100 µm.

The ability of the construct containing the tCUP1 promoter to confer auxin induced DR5::GUS expression was confirmed in T_1_ progeny of both a3-DR5 and c3-DR5. Despite the difference of specific GUS expression in the main root tips of a3-DR5 (root cap, quiescent center and xylem, Fig. 7A) and c3-DR5 (root cap and xylem, Fig. 7E), similar strong and constitutive expressions were induced by exogenous application of auxin NAA for 24 h, a time long enough to promote the initiation and growth of root hairs in the region of the root tip (Fig. 7B and 7F). Increased and expanded GUS expression was also found in the entire lateral roots of a3-DR5 (Fig. 7D) and c3-DR5 (Fig. 7H) as compared to its restricted expression in lateral root cap only (Fig. 7C) and lateral root cap with xylem (Fig. 7G) before treatment. This experiment clearly demonstrated that the tCUP1 promoter used for selectable marker expression did not affect the ability of the DR5 promoter to respond to auxin.

## Discussion

Recently a comprehensive study of strategies to mitigate the nonspecific expression of transgenes was conducted in *Arabidopsis*, demonstrating that the replacement of 35S with tobacco cryptic constitutive promoter (tCUP) was superior to other tested methods in eliminating nonspecific interactions between transgene promoters [14]. We subsequently applied the tCUP derived promoter (tCUP1) to direct selectable marker gene expression and successful transformation in rice [22]. In this study we step further to explore its ability to protect specific transgene expression in rice.

Based on a promoterless reporter system developed in a pCAMBIA backbone, we investigated the potential leaky activity of the tCUP1 promoter compared to 35S and Nos in combination with different configurations (Fig. 1). As expected, when 35S was placed near the GUS reporter in head to head orientation (B configuration), strong activation of GUS expression was observed due to the 35S enhancer (Fig. 2b1). Although such interference could be reduced by inserting a small fragment between the 35S and GUS reporter gene (Fig. 2c1), nonspecific activation was still substantial when combined with the synthetic auxin responsive promoter DR5 (Fig. 6Ai and 6D). This was consistent with the result from mis-expression of the seed-specific napin promoter constructed in the pCAMBIA1391Z vector which has a similar spacer and structure as that of c1 [14]. As the currently used DR5::GUS lines in rice were mainly constructed in pCAMBIA1391Z as previously reported by Scarpella et al. [43], [46], [47], [49], mis-expression of DR5 in these lines could happen especially in the root stele as it does in c1 vector [46], [47]. Interestingly, when the 35S and GUS reporter were separated by the selectable marker gene itself, simply by stacking the two expression cassettes in head to tail orientation (A configuration), specific expression of DR5::GUS in columella cells occurred in some individuals (Fig. 5D and 5E). This result strongly indicated a lower capacity for nonspecific activation by 35S in A configuration, suggesting that the distance between DR5 promoter and 35S enhancer is important for the specificity of DR5::GUS expression.

In contrast to 35S, a low GUS leaky expression was observed with the tCUP1 promoter in both A and C configurations (Fig. 3B). This was not only due to its weak promoter activity in rice leading to low transformation efficiency, but also due to its specific promoter property and the vector configuration, because its relative GUS leaky percentage was still very low after normalization with the spot numbers obtained by vectors in D configuration which represent the transformation efficiency of each promoter (Fig. 3C). Accordingly, the specificity of the DR5 promoter in the root tip was increased by using both vectors, but discernible differences were observed between the two patterns. In a3-DR5 transgenic lines, DR5::GUS seemed to express in the root cap, quiescent center and extending to the protoxylem of the meristem zone (Fig. 6Bc and 7A), quite similar to the typical pattern reported in *Arabidopsis* [50]–[52]. In c3-DR5 lines, DR5::GUS seemed not to be expressed in the quiescent center and vascular initials, but in xylem cells extending to the differentiation zone (Fig. 6Be and 7E). These minor differences probably result from the interlaced spacer as we also found a similar pattern when using c2-DR5 to transform Nipponbare, but more experimental evidence needs to be obtained. Despite the absence of expression in the quiescent center, the auxin inducible activity of c3-DR5 was not affected, as shown by the staining of all the cells in the root tip after addition of the auxin NAA (Fig. 7F). This is in agreement with the conclusion that within the root meristem DR5 actively monitored auxin levels in a cell type independent manner [35].

Since detailed studies with the DR5 reporter are very limited in rice, our results were analyzed largely by comparison with those from *Arabidopsis*. In the roots of *Arabidopsis*, DR5::GUS was most intensely expressed in the columella initial cells and less intensely in the quiescent center and mature columella cells, but was almost invisible in xylem cells as indicated by the GUS reporter [35], [40], [51]. This classical expression pattern was achieved by the DR5::GUS reporter originally constructed in the pBIN19 binary vector by Ulmasov et al. (1997) [39]. The pBIN19 vector contains a selectable marker gene driven by the nopaline synthase promoter near the right border [53]. Therefore the complete DR5::GUS vector has a similar structure to our a2-DR5 in terms of the relative orientation. Unfortunately, we have not found the similar expression pattern in a2-DR5 transgenic lines. Although low leaky GUS expression was observed using the a2 vector without the DR5 promoter (Fig. 3C), it remains to be determined whether the additional distance between the two transcription cassettes in the pBIN19 vector would increase the specificity of the a2-DR5 vector, where they were stacked without an interval.

Auxin distribution in the roots of *Arabidopsis* was also examined using the DR5 variant named DR5rev which consists of nine reversed repeats of the auxin-response element fused to fluorescent proteins [38]. In those studies, DR5rev controlled reporter gene expression was clearly demonstrated by the strong fluorescent signal in the quiescent center, columella and single cell layer of immature xylem cells in the root apical meristem [36], [50], [52]. The enhanced sensitivity of these DR5rev fluorescent reporter constructs even allowed visualization of progressively weaker expression in protoxylem cells from elongation to differentiation zones [36]; that pattern was usually not detectable using the DR5::GUS vector after a short time of staining, but was present after prolonged incubation [35]. Excitingly, we found that this specific pattern could be obtained by the a3-DR5 vector which used the tCUP1 promoter for selectable marker gene expression. Strong and rapid blue staining was shown in the root cap, quiescent center and single cell layer of immature xylem cells in the root apical meristem (Fig. 5F, 5G and 7A), quite similar to that observed by DR5rev fluorescent reporters, except that the lateral root cap cells surrounding the columella cells were also stained blue. This quick response of the a3-DR5 vector to auxin was also evident by comparison with a1-DR5 where maximum blue staining was restricted to columella cells even after a long time of incubation (Fig. 5D and 6Ba), while very weak expression in the quiescent center was found occasionally in some lines (Fig. 5E). This would suggest lower and saturated auxin sensitivity in roots of these a1-DR5 lines, even below the threshold level for histological detection. Overall, these data indicate that the sensitivity of the DR5 promoter was increased when using the tCUP1 promoter for selectable marker gene expression, which was consistent with our previous results showing that using a weak promoter like tCUP1 to express the *HPT* gene against hygromycin selection enhanced the expression levels of the foreign gene [22].

A novel Aux/IAA-based auxin signaling sensor termed DII-VENUS was recently used to map auxin distribution in *Arabidopsis* [50], [51], [54]. The high spatial resolution of DII-VENUS allowed visualization of the distribution of auxin in the entire root tip, with maxima at the quiescent centre and in the columella cells, and minima observed in the epidermis and cortex, while auxin levels significantly increased in the epidermis and vasculature starting at the transition zone between the meristem and the elongation zone [50]. Auxin accumulation in these regions sounds reasonable as there is an ‘auxin reflux’ loop at the root tip mediated by PIN efflux proteins, including lateral auxin redistribution and basipetal auxin transport to the meristem [40]. Such a circulation of auxin is critical for meristem size regulation. In accordance with the auxin distribution revealed by DII-VENUS, we often found patterns of increased GUS expression in the zone of elongation besides distinct expression in the root tip (Fig. 5C; Fig. 6Ab, 6Ad and 6Af), regardless of vector type used, except for c1-DR5 (Fig. 6D). Moreover, this changeable pattern could be detected in different adventitious roots of the same plant and in different T_1_ progenies from the same parent line. Hence we consider that this was a real response to auxin rather than unintended effects from the vector. We also found different patterns in seedlings of different age. Thus in the early primary roots of a3-DR5, clear expression of DR5::GUS was found in the root cap, quiescent center and immature xylem cells as shown in Figure 7A, similar to the pattern in adventitious roots in Figure 5F. In roots that developed later, expression was mainly in the root cap and weak in quiescent center and xylem cells as shown in Figure 6Ac. The dynamic changes in DR5::GUS expression may be influenced by the developmental signals that regulate auxin dependent cell activity and specification, or environmental cues that induce changes of auxin distribution in roots, such as gravity, temperature and pH of the culture medium.

In summary, our work provides a clear profile of distal auxin distribution in root tips of rice by using a DR5::GUS reporter and suggests that a common mechanism of auxin polar transport and responsiveness is present in rice and *Arabidopsis*. Such specific DR5::GUS expression was mainly obtained by use of the tCUP1 promoter to drive selectable marker gene expression, which showed the great ability of the tCUP1 promoter to elevate both the quality and quantity of transgene expression in rice. Therefore it can be a useful vector system for high and specific transgene expression. The transgenic DR5::GUS lines or other endogenous auxin responsive promoter reporters produced by the tCUP1 vector will be valuable tools to understand the exact role of auxin in rice growth and development.

## Materials and Methods

### Plasmid Construction

For acceptance of different promoters to drive *HPT* gene expression in different configurations, a promoterless *HPT* coding region and CaMV 35S terminator fragment was amplified from pCAMBIA1300 by using primers HPT-T35S-F1 (*Asc*I added) and HPT-T35S-R1 (*Sac*I added) for cloning in configuration A with *HPT* near LB (Fig. 1A) or primers HPT-T35S-F2 (*Sac*I-*Asc*I added) and HPT-T35S-R2 (*Mlu*I added) for cloning in configuration B, C and D with *HPT* near RB (Fig. 1B, 1C and 1D). The PCR products were ligated with pMD19-T vector (Takara, Dalian, China) and digested by *Asc*I and *Sac*I or *Mlu*I and *Sac*I; the resulting fragments were subcloned between the *Asc*I and *Sac*I sites of a modified pCAMBIA0380 with multiple cloning sites (MCS) only, resulting in two intermediate HPT-T35S vectors. A GUS (*uidA*) coding region and the nopaline synthase (Nos) terminator fragment was amplified from vector pBI101.3 (Clontech, Palo Alto, CA) by using primers GUS-F1 and TNos-R1 after abolishing the original *Sac*I site by blunt-ending, filling and self-ligation. Multiple cloning sites were added before ATG by using a GUS-MCS primer. The resulting fragment was digested by *Sac*I and *Hind*III and subcloned into the same sites of the two intermediate HPT-T35S vectors to generate HPT-T35S-GUS-TNos and T35S-HPT-GUS-TNos as basal vectors for configurations A and B. To generate the basal vector for configuration D, the same GUS-TNos fragment was amplified by primers GUS-F2 and TNos-R2 with exchanged *Sac*I and *Hind*III sites and the digested fragment was then inserted into the T35S-HPT intermediate vector with GUS reporter near RB. To generate the basal vector for configuration C, a spacer sequence was amplified from pCAMBIA1300 between 35S for hygromycin phosphotransferase gene expression and multiple cloning sites by primers Spacer-F and Spacer-R, and was introduced into the *Sac*I and *BamH*I sites of the basal vector of configuration B. The 35S, Nos and tCUP1 promoter fragments were obtained as previously described [22] and inserted before the HPT-T35S cassette in each basal configuration vector in forward orientation. All the primers used are listed in Table S1 and the sequences of the spacer, 35S, Nos and tCUP1 promoters are provided in Table S2.

The heptadic repeat of the synthetic auxin responsive DR5 element [39] coupled to the −46 CaMV 35S minimal promoter and TMV omega enhancer was amplified by primers DR5-PF and DR5-PR with introduced *Sal*I and *Kpn*I sites for cloning into the MCS of GUS reporter vectors a1–a3 and c1–c3 (Fig. 4). The sequence of a3-DR5 is provided in Table S3. The constructs and related transgenic lines are available to the research community upon request.

### Plant Material and Transformation

The binary vectors described above were introduced separately into the *Agrobacterium tumefaciens* strain EHA105 by electroporation and transformed to embryogenic calli developed from mature seeds of rice by the method previously reported [22]. The *japonica*-type rice *O.sativa* L. cv. Nipponbare was used for the study of the leaky GUS expression. After the first round of selection (21 days), 50 calli from each transformation with GUS reporter vectors indicated in Figure 1 were selected and stained. Cultivar Ishikari-shiroge (I–S) was used for stable transformation to obtain DR5::GUS transgenic lines.

### Growth Conditions and Auxin Treatment

Regenerated rice T_0_ transgenic plants were grown in International Rice Research Institute (IRRI) rice culture solution in a growth room with 30°C/25°C (day/night) temperature and 60%–70% humidity under a 12 h photoperiod until harvesting of T_1_ seeds. Geminated T_1_ seeds were placed in a plastic net floating in rice culture solution with added 0.5 mM MES to stabilize pH at 5.2. Primary roots (from root tip to the region with lateral roots) of 8-day-old seedlings were sampled for histochemical GUS assay. For auxin treatment, 8-day-old c3-DR5 seedlings were moved to rice culture solution with or without 1 µM NAA and treated for 24 h; 6-day-old a3-DR5 seedlings were treated under the same condition. Primary roots were sampled from treated and mock treated seedlings and stained in 1 mM X-gluc for 30 min.

### Histochemical GUS Assay and Microscopic Observation

Histochemical detection of GUS activity was performed as described by Jefferson et al. (1987) [55]. Transformed calli after 21-days selection were incubated in 100 mM phosphate buffer (pH 7.0), 10 mM sodium EDTA, 1 mM potassium ferricyanide, 1 mM potassium ferrocyanide, 20% (v/v) methyl hydrate, 0.5% (v/v) Triton X-100, 0.5 mM 5-bromo-4-chloro-3-indolyl-β-D-glucuronide (X-gluc) at 37°C for 24 hours. Calli were viewed with a Nikon SMZ1000 stereomicroscope and images were acquired with a Nikon digital camera DS-Fi1. Adventitious roots of 1-month-old T_0_ plants were dissected and incubated at 37°C in staining solution containing 1 mM X-gluc for 1 h or 0.5 mM X-gluc for 4 h. Primary roots of 8-day-old seedlings of transgenic T_1_ plants were incubated in 1 mM X-gluc for 30 minutes (a2-DR5, a3-DR5, c1-DR5, c2-DR5, and c3-DR5) or 16 hours (a1-DR5). The staining solution was removed with water and tissue was infiltrated under vacuum for 4–5 periods of 30 s (Eppendorf, Concentrator Plus, Mode: D-AQ, 30°C). The water was replaced with enough chloral hydrate/glycerol solution (1.6 g chloral hydrate to 1 ml 20% glycerol) to cover the tissue which was allowed to clear for several hours. Cleared samples were mounted in chloral hydrate/glycerol solution under a coverslip and directly viewed with a Nikon Eclipse Ti inverted DIC microscope imaging system.

## Supporting Information

Table S1 Sequences of the primers used in this study.
(DOC)

Table S2 Sequences of spacer, 35S, Nos and tCUP1 promoters.
(XLS)

Table S3 Vector sequence of a3-DR5.
(XLS)
