# Supplementary material for: Specific Expression of DR5 Promoter in Rice Roots Using a tCUP Derived Promoter-Reporter System
Source: PLoS One. 2014 Jan 22;9(1):e87008. doi: 10.1371/journal.pone.0087008 (PMC3899362; doi:10.1371/journal.pone.0087008)
Supplement: Table S1 — Sequences of the primers used in this study. (DOC) [file pone.0087008.s001.doc]

**Supporting information**

**Table S1** Sequences of the primers used in this study.

Primer Sequence (5’-3’)^a^
tCUP-PF GGCGCGCCATCTTCTGCAAGCATCTCTATTTCC
tCUP-PR GGCGCGCCCCATGGTGGCCGGTGGGTTT
35S-PF AGGCGCGCCGGTCCCCAGATTAGCCTTTTC
35S-PR AGGCGCGCCGTCCCCCGTGTTCTCTCC
Nos-PF AGGCGCGCCGATCATGAGCGGAGAAT
Nos-PR AGGCGCGCCAGATCCGGTGCAGATTA
Spacer-F AGAGCTCTGCTCCACCATGTTGGCAAG
Spacer-R AGGATCCGTAATCATGGTCATAGCTGTTTC
HPT-T35S-F1 AGGCGCGCCATGAAAAAGCCTGAACTCAC
HPT-T35S-R1 TGAGCTCTAATTCGGGGGATCTGGATTTTAG
HPT-T35S-F2 AGAGCTCGGCGCGCCATGAAAAAGCCTGAACTCAC
HPT-T35S-R2 TACGCGTTAATTCGGGGGATCTGGATTTTAG
GUS-F1 ATGGTAGATCTGAGGGTAAATTTC
GUS-F2 AAAGCTTCCGGGGATCCTCTAGAGTCG
TNos-R1 AAGCTTCCCGATCTAGTAACATAGATGA
TNos-R2 AGAGCTCCCCGATCTAGTAACATAGATGACACC
GUS-MCS GAGCTCCCGGGGATCCTCTAGAGTCGACGAATTCGGT
 ACCATGGTAGATCTGAGGGTAA
DR5-PF GGTCGACGGTATCGCAGCC
DR5-PR TGGTACCTCCCTGTAATGTAAATAGTAATTG

^a^Restriction sites added at 5’ end of primers are underlined
